# Medicine

**Published:** 1897-09

**Authors:** J. Michell Clarke


### Progress of the Medical Sciences.

### MEDICINE.

The proved value of **thyroid extract** in myxœdema and other conditions has led to numerous researches into the nature of the active principles of the gland. Kinnicutt states[1] that "Baumann has isolated an iodine-bearing compound of albumin and globulin, which he has named 'Iodothyrine,'" and believes to constitute the active principle of the gland. R. Hutchison finds[2] that "the thyroid contains two proteids—a nucleo-albumin and the colloid matter"; the former is small, the latter large, in amount. The colloid contains a little phosphorus and much iodine, and is the only specifically active constituent of the gland. Both the non-proteid and the proteid parts of the colloid are active; but the former (which is apparently identical with Baumann's iodothyrine) is the more so.

**How does thyroid extract affect the metabolism of the body?** L. A. Gluzinski and Ig. Lemberger[3] obtained the following results in a healthy man: for a preliminary period of seven days an accurate estimation of the constituents of the food ingested, and of the excreta, was carried out, until nitrogenous equilibrium was established, with a slight gain in body-weight and retention of nitrogen (N. balance positive). Then during the administration of thyroid tabloids for six days there was a slight diminution of weight—due to loss of fat and water, and not to any excessive disintegration of nitrogenous tissues. They found that thyroid extract, however, exerts an accelerating influence on nitrogenous tissue-change, this influence increasing with the duration of its administration. When, after an interval of six days, *fresh* calf's thyroid was given, during a second period of four days, in about the same doses as the tabloids, this preparation had a much more energetic action, causing a more rapid loss of weight; and in this process of disintegration the nitrogenous tissues shared, as shown by the increased loss of N., which exceeded the N. intake (negative N. balance). This loss of weight continued, though with less degree of nitrogenous tissue-change, for three days after the administration had been stopped. It is important to note the more energetic action on the nitrogenous tissues of the fresh gland than that of the tabloids. These facts offer a probable explanation of some of the discrepancies in the results obtained by other observers. R. David,[4] who administered large doses of thyroid tabloids,

1 *Am. J. M. Sc.*, 1897, cxiv. 1. 2 *J. Physiol.*, 1896, xx. 474.
3 *Centralbl. f. innere Med.*, 1897, xviii. 89.
4 *Ztschr. f. Heilk*, 1896, xvii. 439; abstract in *Centralbl. f. innere Med.*, 1897, xviii. 565.

found that the nitrogen excreted in the urine was much increased and that this increase continued after the drug was stopped, and then suddenly ceased. Coincidently there was loss of weight, diuresis, no difference in the amount of uric acid, but increase in that of phosphorus excreted. In a case of myxœdema treated by iodothyrine for six days, the patient lost 1.9 kilos. in weight in ten days, this being attended with an increased elimination of nitrogen. So also Irsai, Vas, and Gara[1] found in three cases of goître treated with thyroid tabloids a diminution in the size of the goître; a decrease in the body-weight, the loss varying in amount with the duration of treatment; an increase in the quantity of urine and in the nitrogen excreted, and also in the excretion of sodium chloride and phosphoric acid. On these results the treatment of obesity by thyroid extract is based. "In patients who react to treatment, the loss of weight varies from 2—11 lbs. a week, without restriction of diet; but after a time a limit is reached, beyond which a further reduction is not obtained. The loss of weight is more rapid during the first days of treatment than later; subsequently it varies in amount from week to week. . . . The loss is usually unattended with any disturbance of health. As a rule, the discontinuance of treatment is quickly followed by an increase of weight, which shortly reaches its former level. Only exceptionally is the improvement maintained for any considerable period, although careful dieting and exercise prolong its duration."[2]

In **cretinism** the success of the thyroid treatment has been remarkable. The results attained are, according to Dr. Rushton Parker:[3]—(1) Absorption and disappearance of the various myxœdematous deposits and swellings. (2) Great and rapid increase in physical development, shown especially by a rapid growth of several inches in height, even in cretins of 20—30 years of age, and an increase in the body-weight. (3) Diminution of such deformities as spinal lordosis, bulkiness of head, depressed nose, &c. Owing to the softening of the bones produced by thyroid extract, there may, however, be increased deformity (bowing) of the legs. (4) A rapid and striking increase of intelligence. Dr. John Thomson, in the same discussion, showed that the rate and amount of the increase in height is in inverse ratio to the age of the patient and to the stage of the treatment. Thus children grow more than adolescents, and adolescents more than adults: the rate of growth is at first very rapid, but becomes slower as the height approaches that of the normal for the age. He also notices the tendency to spinal curvature and bowing of the legs, and thinks that mental improvement is not distinct until the sixth or eighth month of the treatment, and forms part of a general amelioration. Dr. Telford Smith said that he had given thyroid extract to idiots

[1] *Deutsche med. Wchnschr.*, 1896, xxii. 439. [2] Kinnicutt, *loc. cit.*

[3] "A Discussion on Sporadic Cretinism in this Country and its Treatment." *Brit. M. J.*, 1896, ii. 615.

belonging to the so-called Mongol type, who most nearly resemble the cretin, and found both physical and mental improvement, which varied inversely as the age of the patient, but was not so marked or so rapid as in the case of cretinism. It is advisable to employ small doses in these cases.

Thyroid extract has been successfully employed in **insanity**. Dr. C. G. Hill[1] has treated forty cases, consisting chiefly of commencing senile dementia, acute mania and melancholia, of whom eight were unaffected by the treatment, twelve were somewhat, fourteen very much, improved, five cured, and one died. He states that the drug had an altogether extraordinary influence on the mental condition of the patients. Amongst clinical symptoms during the use of the remedy, rise of temperature and pulse-rate, gastric disturbances, increased perspiration and quantity of urine, transient albuminuria in 10°/₀, œdema of face and extremities, cyanosis, desquamation of the skin, sexual excitement, so that masturbation in three cases necessitated the discontinuance of the thyroid extract, were observed. Of sixty cases treated by Bruce, there were twenty-four recoveries; and in twenty-two cases reported by Babcock, four recovered and eleven improved. Kinnicutt[2] says that Allen Starr places "the highest value upon the treatment in a form of mental disturbance occurring at the climacteric. He describes it as a mental depression with anxiety and morbid fears, but without delusions or insomnia." Clouston says "that large doses of thyroid are of marked benefit in cases which threaten to become chronic, and that no case should be allowed to become incurable without a trial of this method of treatment." Dr. Henry J. Berkeley[3] treated eight insane patients with thyroid tablets, beginning with one daily, and increasing to three if no grave symptoms appeared. In addition to loss of weight, tachycardia or feebleness of cardiac action, slight pyrexia, digestive disturbances, and increased perspiration, irritability and a greater or less degree of mental and motor excitement were remarked in all cases, no matter how depressed or demented they had been previous to the administration. Two patients became frenzied. Of the eight patients one recovered, one died of acute miliary tuberculosis, in the others there was no improvement. Dr. Berkeley concludes that the administration of even the best and purest thyroid tablets "is not unattended with danger to the health and life of the patient, and . . . may be followed by symptoms not only difficult to control, but of very marked influence upon the future mental powers of the subject." He also administered thyroid tablets to five mice and three guinea-pigs; the lethal dose in the latter could be exactly estimated, and was found to be from 1 to 2 °/₀ of the body-weight. Examination of the cerebra of these animals by the most approved

[1] *Tr. M. & Chir. Fac. Maryland*, 1896, p. 30; abstract in *Centralbl. f. innere Med.*, 1897, xviii. 566.

[2] *Loc. cit.* [3] *Johns Hopkins Hosp. Bull.*, 1897, viii. 137.

histological methods failed to show any morbid changes; the poison corresponds in its action to that of a group of chemical poisons that leave no trace of their effect after death upon the nerve cell, but during life induce symptoms directly referable to the central nervous system.

Many observers report good results from the administration of thyroid extract in **goître**; it appears to be most efficacious in simple hyperplastic goître, the cystic form being unaffected, except for a shrinking of the parenchymatous tissues surrounding the cysts. Diminution in size rather than complete disappearance of the tumour is the general result.

In **skin diseases** thyroid extract has been used with some success in the treatment of psoriasis; in other affections of the skin it does not appear to be of value.

Dr. Kinnicutt[1] has collected forty-eight cases of **Addison's disease treated with various preparations of the suprarenal gland.** Six are reported as cured or well, twenty-two improved, eighteen unimproved, and in two an aggravation of the symptoms occurred. Of the six recoveries, in one the patient has remained well for four months; in another the patient was well eighteen months after the beginning of the treatment, and has been able to return to work, and in a third the patient remained well twelve months afterwards. Of the cases in which improvement was reported, relapse and death occurred in two, and relapse in a third, in which the treatment was irregular. In a fourth treatment was suspended after a month, on account of failure of appetite, and the patient died. In a fifth there was improvement for three months, when sudden death occurred on giving up the treatment. Another patient improved during regular treatment, but fell off rapidly during the subsequent eight months when it became irregular. In another, improvement was maintained for twelve months, and work was resumed, but death occurred at the end of this time. Relapses occurred in one case when the treatment was suspended. In a woman under my care with all the classical symptoms of Addison's disease, who was so weak that she was obliged to keep her bed on admission to the Hospital, remarkable improvement resulted from two months treatment, the pigmentation disappeared, and she returned to her work as a laundress; but, unfortunately, I could not trace her after she left the Hospital, so that I do not know the ultimate result. A daily dose equivalent to 45 grains of the fresh gland is recommended.

Drs. Abel and Crawford[2] conclude, from their researches into the constitution of the blood-pressure-raising constituent of the suprarenal capsule, that it is to be classed with the pyridine bases or alkaloids, and that it is not possible to split off pyrocatechin from the isolated active principle. Mühlmann

[1] *Loc. cit.*

[2] *Johns Hopkins Hosp. Bull.*, 1897, viii. 151.

supposed that the blood-pressure-raising constituent is pyrocatechin joined to some other substance, probably an acid; but according to Abel and Crawford this is not the case.

On account of its remarkable power of raising the blood-pressure, the administration of suprarenal extract has been advocated in those cases of **anæmia and neurasthenia** in which there is a loss of vaso-motor tone.

Dr. Solis-Cohen[1] has given **suprarenal extract in exophthalmic goître.** In two cases, not of severe type, the tremor and cardio-vascular phenomena were improved; in another marked case a similar result was gained; a fourth patient was made worse. Compared with thymus the suprarenal extract seemed to have a greater influence upon the circulation and less effect upon the goître and exophthalmos.

Svehla[2] has made a series of experiments on dogs with dried and watery extracts of the **thymus** from bullocks, pigs, dogs, and man. The effect on the circulation was chiefly studied. He concludes that injection of thymus extract (into the femoral vein) is followed by a fall of blood-pressure due to weakening or paralysis of the vaso-constrictors, and increase of pulse rate, due to direct influence on the heart. When large doses were given there was excitement, followed by dyspnœa and collapse, ending in death, with *post-mortem* appearances of asphyxia. Hence it is possible that some of the so-called cases of "thymic asthma" in children may be due to an excess of thymic secretion in the blood.

The use of **thymus extract in exophthalmic goître** appears to have followed from the good effects obtained in one case by Mr. David Owen, of Manchester, in which the butcher supplied the patient with thymus instead of thyroid gland. Dr. Hector Mackenzie[3] has made a careful investigation into the value of thymus extract in this disease. Of fifteen cases collected by him from the literature of the subject, fourteen were reported as decidedly improved. His personal observations amount to twenty patients treated by thymus extract, of whom one died, six remained the same, and thirteen were somewhat improved. In no case however did the extract exhibit such a distinctive influence on the most important symptoms, and on the course of development of the disease as to be recognisable as a specific remedy for it. He also treated by the usual methods, for comparison with the foregoing, twenty other cases presenting an approximately similar condition of pulse, goître and exophthalmos. His conclusion is that only the general condition of the patient was improved by the thymus treatment. The size of the dose showed no marked effect. Dr. Solis-Cohen[4] reports improvement in six out of twelve cases of exophthalmic goître

[1] *J. Am. M. Ass.*, 1897, xxix. 65.
[2] *Wien. med. Bl.*, 1896, xix.; abstract in *Brit. M. J.*, 1897, i. Epitome, p. 88.
[3] *Am. J. M. Sc.*, 1897, cxiii. 132. [4] *Loc. cit.*

in which he had administered thymus extract. Goître, exophthalmos, palpitation were improved, and nervousness, insomnia, and tremor very much relieved. Maude[1] has published four cases which received benefit, but he does not think that the results attained were greater or more permanent than they might have been under other treatment or none at all. Kinnicutt[2] gave a careful trial of the thymus to two patients for two months, but without any beneficial effect upon any symptom. One of the cases improved subsequently under belladonna and bromides.

On account of the apparent influence of thymus extract on the general nutrition, Dr. Kinnicutt also treated six selected cases of **apyrexial pulmonary phthisis** with doses of 45—60 grains daily, given continuously for two months. In five of the six cases an increase of weight occurred during the first weeks of treatment. The gain was followed by a loss varying in amount. In only one case was there no loss in weight during the two months of the thymus treatment. In all the cases there was little or no change in the general condition and in the physical signs.

Dr. Kinnicutt[3] has collected thirteen cases of **acromegaly treated with pituitary gland** preparations. In seven cases varying degrees of improvement were noted: in one of them under the combined use of pituitary and thyroid extract; in five cases there was no effect; and one patient was made worse. Improvement took place in the general condition, and in the headache and neuralgic pains.

Dr. H. C. Wood[4] has treated **exophthalmic goître by splenic extract**, the treatment being based on the complete recovery of a case of chronic exophthalmic goître after an acute attack of splenitis, with subsequent discharge of an abscess of the spleen. A good result was obtained in one case, but there is some difficulty in giving the extract, as sufficient doses by the mouth are apt to cause dyspepsia, pain, and vomiting, and hypodermically to produce great irritation locally.

J. MICHELL CLARKE.

### SURGERY.

[1] *Lancet*, 1896, ii. 173. [2] *Loc. cit.* [3] *Loc. cit.*
[4] *Am. J. M. Sc.*, 1897, cxiii. 511.

